# Farm Houses

**Published:** 1866-04

**Authors:** 


### HALL'S JOURNAL OF HEALTH.

Vol. XIII.] APRIL, 1866. [No. 4.

### FARM HOUSES, CONTINUED.

In building a new house, or in remodeling an old one, the upper rooms, the chambers especially, when practicable, should be so arranged that the sun should shine into them through windows on three sides; this would afford admirable facilities for ventilation, and would give the light, and dryness, and cheerfulness, which so much contribute to the healthfulness of a chamber, and the lively, cheerful temper of those who occupy them. All farm-houses should be arranged, as far as possible, so that the rooms which are to be most generally occupied should have most of the sun during the day. It is too often the case that the parlor, the company room, is the largest, lightest, and best room in the building; this parlor is barricaded with curtains, window-shutters, and closed doors, except when there is "company," which will, perhaps, average about a dozen half days in the year; the remainder of the time all its sweetness is

"Wasted on the desert air."

By all means let the best room in the house be enjoyed every day by the members of the family; give the room which is largest and lightest to your own wife and children all the time, instead of saving it for other people for a dozen hours in the year. Besides, such a room, almost always closed up, is a positive injury to every person who enters it; for in winter it has a pernicious "closeness" about it, while in summer there is a mustiness and dampness, often a "chilliness" present, which makes it feel almost sepulchral the moment it is entered.

